# Development and Feasibility of a Smartphone Application for Promoting Healthy Heart Behaviors Following Open-Heart Surgery: A Mixed-Method Pilot Study

**DOI:** 10.3390/healthcare13141647

**Published:** 2025-07-08

**Authors:** Preeyaphorn Songsorn, Pawarat Nontasil, Kornanong Yuenyongchaiwat, Noppawan Charususin, Jitanan Laosiripisan, Sasipa Buranapuntalug, Khanistha Wattanananont

**Affiliations:** 1 Physiotherapy Department, Faculty of Allied Health Sciences, Thammasat University, Pathum Thani 12120, Thailand; kornanong.y@allied.tu.ac.th (K.Y.); noppawan.c@allied.tu.ac.th (N.C.); jitanan.l@allied.tu.ac.th (J.L.); sasipa.b@allied.tu.ac.th (S.B.); 2 Thammasat University Research Unit for Physical Therapy in Respiratory and Cardiovascular Systems, Thammasat University, Pathum Thani 12120, Thailand; 3 National Electronics and Computer Technology Center (NECTEC), Pathum Thani 12120, Thailand; pawarat.nontasil@nectec.or.th; 4 Cardiac Rehabilitation Center, Faculty of Medicine Vajira Hospital, Navamindradhiraj University, Bangkok 10300, Thailand; khanistha@nmu.ac.th

**Keywords:** cardiac rehabilitation, telemedicine, mobile applications, postoperative care, health behavior

## Abstract

**Background/Objectives:** Adherence to healthy behaviors after open-heart surgery is crucial for recovery and long-term health. Traditional patient education methods can be enhanced by using technology to improve engagement and self-care. This study aimed to develop and assess the feasibility of the “Term-Jai” smartphone application for promoting healthy heart behaviors in open-heart surgery patients. **Methods**: The “Term-Jai” psychological theory-based application was tested quantitatively and qualitatively over a 30-day period with 13 patients (age 44–78 years) following open-heart surgery between November 2023 and March 2024. Participant engagement, healthy behaviors, user experience, and usability were assessed using the System Usability Scale (SUS), satisfaction ratings, healthy behavior questionnaires, and semi-structured interviews. **Results**: The application was feasible, with 70% of participants remaining engaged during the intervention. The average SUS score was 80.2 ± 10.3, indicating good usability. Participants found the application’s information useful, clear, and easy to understand, showing improvements in health behaviors following application usage. The qualitative analysis highlighted the application’s intuitive design and potential for supporting cardiac rehabilitation. High satisfaction scores suggested its effectiveness despite some barriers to application usage around technical support and personalized exercise progression. **Conclusions**: The “Term-Jai” application is a promising tool for promoting healthy behaviors in patients following open-heart surgery. The application shows good usability and participant satisfaction, indicating its potential for broader implementation after further refinements.

## 1. Introduction

According to data from the World Health Organization, coronary artery disease was identified as the leading cause of death worldwide [1]. In Thailand, the Ministry of Public Health’s statistics for 2021 show that, nationally, coronary artery disease is the fourth leading cause of death [2]. Treatments for coronary artery disease include medication, lifestyle adjustments, and interventions like Percutaneous Coronary Intervention (PCI) or Coronary Artery Bypass Surgery (CABG) [3]. Cardiac rehabilitation (CR) following these procedures is critical to reducing mortality and rehospitalization rates [4,5,6]. Despite its benefits, only approximately 18.3% of eligible patients in Thailand attend CR [7]. Barriers to accessing CR services in Thailand include a lack of knowledge regarding CR and its benefits, transportation issues, CR-related costs, and access to the services [8]. Ragupathi L. and colleagues suggest that utilizing mobile health technology (mHealth) for remote health monitoring and communication could help overcome these barriers and improve access to CR services [9].

Mobile health technology (mHealth), a component of electronic health (eHealth), has undergone significant advancements. Its benefits include primary and secondary prevention, personalized treatment recommendations, enhanced communication between healthcare teams and patients, enhanced access to healthcare services, patient engagement in continuous care, and real-time treatment monitoring [10]. Cruz-Ramos et al. noted the diversity of mHealth solutions for cardiovascular disease self-management, such as mobile applications for electrocardiogram monitoring, heart rate tracking, and educational tools promoting healthy behaviors like physical activity and medication adherence [11]. Furthermore, research shows mHealth’s positive impact on patient populations, including those with heart failure, arrhythmias, coronary artery disease, hypertension, and those in CR programs [11,12]. Previous research has consistently demonstrated the effectiveness of smartphone-assisted cardiac rehabilitation in improving clinical outcomes. These studies have shown that the integration of smartphone applications into cardiac rehabilitation programs significantly enhances exercise capacity, increases treatment adherence, promotes healthier lifestyle behaviors, and reduces hospital readmissions, emergency department visits, and postoperative complications [13,14,15,16]. Furthermore, such interventions have been associated with improvements in patients’ quality of life [17]. These findings provide robust evidence supporting the integration of mobile health technologies into cardiac rehabilitation programs, reinforcing their potential to improve patient engagement and adherence in both clinical and home-based settings. This study aimed to develop and evaluate the feasibility of a smartphone application to promote adherence to healthy heart behaviors following open-heart surgery.

## 2. Materials and Methods

### 2.1. Study Design

This study used a mixed-methods design, integrating quantitative and qualitative approaches, to explore the feasibility of a smartphone application to promote adherence to healthy heart behaviors in patients who underwent open-heart surgery. A research assistant provided participants with a brief educational session on application usage. Participants completed a user satisfaction and usability questionnaire and were interviewed by phone after 30 days of application usage.

### 2.2. Application Design

The application aims to encourage users to transition from procrastination to routine behavior and habit formation. Procrastination, defined as a failure of self-control or self-regulation, involves conscious or unconscious decisions that delay task completion [18]. Causes include lack of motivation, poor time management, and negative emotional states, and can result in adverse outcomes such as unhealthy behaviors [19,20].

The application’s design uses the Fogg Behavior Model [21], which states that motivation, ability, and triggers must coincide for a target behavior to occur. Ideally, a target behavior occurs when it requires minimal effort and high motivation. Users are prompted to complete easy daily tasks and are rewarded upon task completion to boost motivation.

### 2.3. Application Development Process

The application was developed between January 2023 and July 2023, using the Analysis, Design, Development, Implementation, and Evaluation (ADDIE) model [22]. The analysis phase involved a comprehensive literature review to identify clinical guidelines, behavior change strategies, and mobile health trends relevant to CR. This was followed by a comparative analysis of existing CR applications to assess common features and user limitations, which informed the application’s functional and educational requirements. The design phase focused on conceptualizing the application’s two main components: promoting daily physical activity through guided exercise and delivering structured health education on cardiovascular disease management. During the development phase, the application was built for the Android platform using the Dart programming language and Flutter framework, following the learning objectives and the structure outlined in the analysis and design phases. The first version of the application was evaluated by three experts (two physiotherapists and one cardiothoracic surgery nurse) with over five years of CR experience. Following revisions, the implementation phase involved distributing the application via smartphone and conducting evaluations post-usage.

### 2.4. Participants

The participants in this study were individuals who underwent open-heart surgery at Vajira Hospital in Thailand between November 2023 and March 2024. Eligible participants were 20–80 years old, had an Android smartphone, and completed the inpatient cardiac rehabilitation phase (Phase I CR program). Participants with cognitive impairment or postoperative complications such as respiratory infections, wound infections, and unstable vital signs, were excluded. This study was approved by the Institutional Review Board of the Faculty of Medicine, Vajira Hospital (COA no.177/2566). The Thai Clinical Trials Registry number is TCTR20231017008. All of the participants in the study provided informed consent. Written consent was obtained from the participants for the publication of this paper.

### 2.5. Feasibility, Data Collection, and Outcome Measures

The feasibility study evaluated the application’s usability, user satisfaction, and engagement. Participants were required to use the application at least five days a week for 30 days. After 30 days of usage, participants completed the questionnaires in a paper-based format. The System Usability Survey (SUS) to assess the application’s usability. The SUS consisted of ten items and was adapted from a previously validated version [23], which scored on a 5-point Likert scale. The final score ranged from 0 to 100, with higher scores indicating better usability. Participants also completed an 8-item satisfaction questionnaire to evaluate the participants’ experience with the contents and design of the application throughout the study. The user satisfaction questionnaire was adapted from a previous research study [24]. Each item was scored on a 5-point Likert-type scale ranging from 1 (not satisfied at all) to 5 (extremely satisfied). The application’s acceptability was further evaluated through semi-structured interviews (Table 1).

Improvements in health behavior were evaluated using a questionnaire adapted from a previously published study [25] which focused on cardiovascular disease risk behaviors. Responses were rated on a scale from 1 (no engagement in the behavior) to 5 (consistent and active engagement). An increase of at least one point was interpreted as an indication of positive change in health-related behavior. Participant engagement with the application was measured by the number of days they used its features for at least 20 out of 30 study days [26].

### 2.6. Data Analysis

#### 2.6.1. Quantitative Data

Descriptive statistics were used to summarize participants’ demographic data, showing means, standard deviations, and proportions.

#### 2.6.2. Qualitative Data

A content analysis of the semi-structured interview transcripts was conducted to assess their usability using thematic analysis [27]. To ensure the accuracy and reliability of the data, the transcripts were rigorously cross-checked against the original audio recordings. Key statements reflecting the experiences and perceptions of participants were identified and systematically coded. Each newly generated code was compared with previously established ones, eventually forming subthemes and overarching themes. Finally, the themes were refined, and the core aspects of the application’s feasibility in practical implementation.

## 3. Results

**Figure 1:**
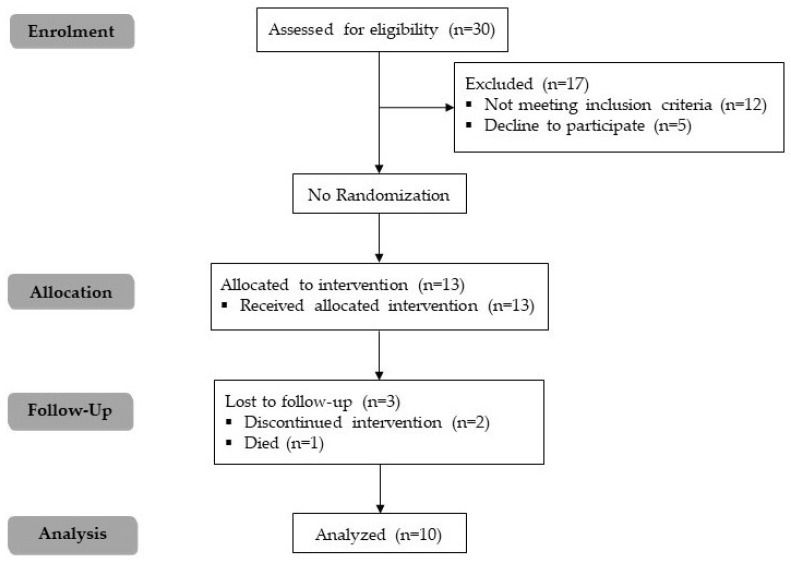
CONSORT flow diagram for a single-group pilot study.

A total of 30 participants were assessed for eligibility, of which 12 did not meet the inclusion criteria, and five declined to participate for personal reasons. As a result, 13 participants were enrolled in the study. Three participants dropped out, two discontinued the intervention for personal reasons, and one died for reasons unrelated to this study (Figure 1).

### 3.1. Development of the Smartphone App

**Figure 2:**
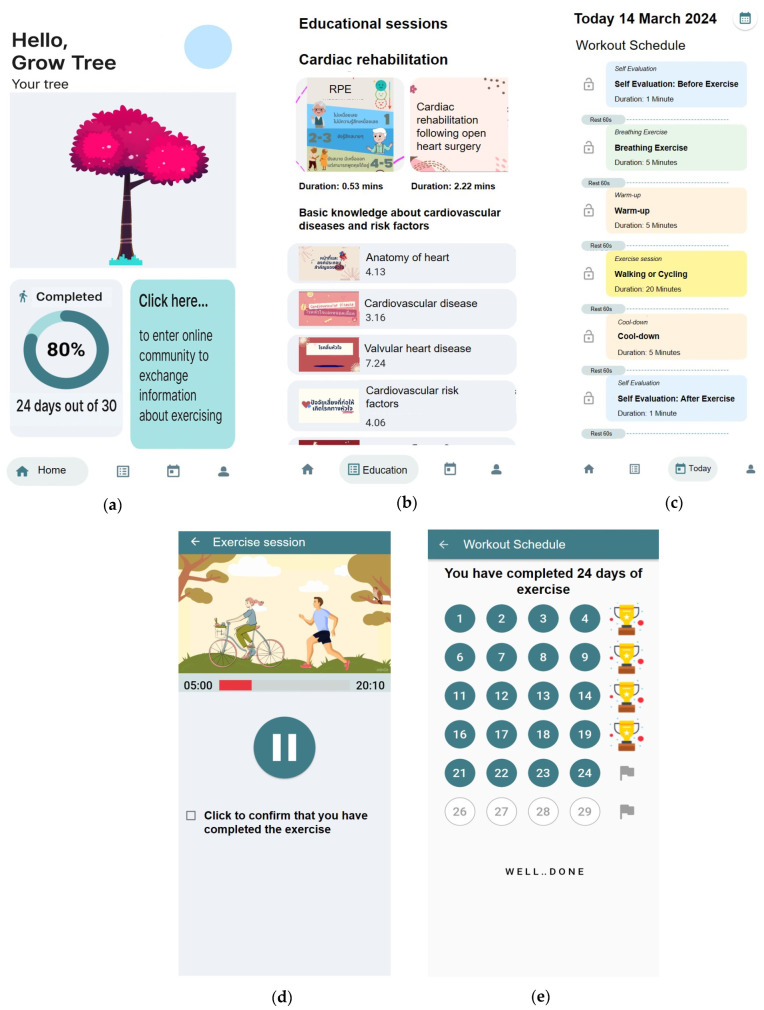
The screenshots of “Term-Jai” smartphone app. (**a**) The home screen displays the user’s profile and exercise progression, including an illustration of a growing tree and a percentage of completion; (**b**) The educational sessions feature video and audio media covering fundamental knowledge about cardiovascular diseases, risk factors, and cardiac rehabilitation (CR); (**c**) The workout section includes today’s session and a summary of exercise activity; (**d**) exercise session; (**e**) green circle appears each day upon exercise completion, and users receive a trophy after completing five sessions per week.

The “Term-Jai” (meaning “fulfilled heart” in Thai) application was designed to be user-friendly. According to the ADDIE model, the analysis phase identified key limitations in previous apps, including a lack of guided exercise content, minimal user engagement features, and limited educational support. These findings informed the definition of user needs and learning objectives for the application, emphasizing simplicity, motivation, and comprehensive CR support. Based on this analysis, the application was designed with two primary components: a daily exercise module and a set of educational sessions. These components include three main features: a daily exercise plan, exercise progress tracking, and educational content. The daily exercise plan involves self-assessment before and after each session using the rated perceived exertion (RPE) scale, along with video guides for breathing exercises, warm-up, main exercise sessions, and cool-down. A visual representation of a growing tree and a green circle appears upon daily completion of the exercise. Participants receive a trophy after completing five sessions in a week, recognizing their exercise progress. The educational sessions incorporate video and audio media covering basic knowledge about cardiovascular diseases, associated risk factors, and CR (Figure 2). The initial prototype was evaluated by experts, whose feedback led to improvements in video quality, text size, clarity of visual instructions, and consistency of the user interface layout. The revised application was then implemented on users’ smartphones for real-world use.

### 3.2. Baseline Characteristics of Participants

The demographic and clinical characteristics of ten participants are shown in Table 2. The average age was 60.4 ± 10.0 years and the average BMI was 25.0 ± 3.5 kg/m^2^. Most of the participants in this study were male (80%). The surgeries performed included coronary artery bypass surgery (50%), valve replacement (40%), and aortic hemiarch replacement (10%).

### 3.3. Quantitative Findings

#### 3.3.1. Application Engagement

70% of participants met the engagement requirement by using the application for at least 20 out of the 30 days. One participant did not use the application regularly because of technical problems, and two participants lost interest in it.

#### 3.3.2. Application Usability

Ten participants completed the SUS after 30 days, with a mean score of 80.2 ± 10.3 (range 70–92.5) out of 100. Table 3 shows the five-point Likert scale scores for the ten SUS questions.

#### 3.3.3. Application User Satisfaction

Table 4 lists the mean values of user satisfaction with the “Term-Jai” application’s content and design. The average participant satisfaction score was 4.3 ± 0.6 out of 5. All participants stated that the application’s information was useful; 90% said it was clear and easy to understand and felt that the content quantity was appropriate. Moreover, all participants stated that the screen color was appropriate, 80% found that the application made learning easy and that the font size was appropriate, and 60% said that the overall composition of the application was appropriate.

#### 3.3.4. Healthy Behaviors Improvement

All participants improved their health behaviors (Table 5), including more exercise, increased consumption of vegetables and fruits, reduced salt intake, and decreased consumption of high-fat and high-sugar foods. They also managed stress better. However, smoking cessation and alcohol consumption remained unchanged.

### 3.4. Qualitative Findings

Based on semi-structured interviews, the participants identified four main themes: appropriate application content, appropriate application design, barriers to use, and suggestions for improvement. Most participants were satisfied with the application content, finding it beneficial, especially for exercise programs and educational sessions. Participants also found that the application was user-friendly. However, some participants reported technical issues, and others offered useful suggestions for improvement. The themes, subthemes, and participants’ interview quotes are presented in Table 6.

## 4. Discussion

This study aimed to develop and investigate the feasibility of the “Term-Jai” smartphone application to promote adherence to healthy heart behaviors following open-heart surgery. Quantitative and qualitative data revealed that the “Term-Jai” application was feasible for improving healthy heart behaviors.

The findings of this study align with existing studies on the development of smartphone applications for patients recovering from cardiac surgery [12,28,29]. Previous studies demonstrated high engagement with applications in peri-operative cardiac surgery patients [28] and patients enrolled in CR [12]. These findings align with the present study, which showed that 70% of participants remained engaged with the “Term-Jai” application over a 30-day intervention. However, the engagement in this study was higher than that in a previous study on cardiovascular diseases [29]. This difference may be due to the variant psychological strategies used in this study, such as gamification strategies, tree growth visualization, green circle visualization, and trophies. Gamification strategies effectively promote behavior change by making health-related activities more engaging and motivating participants to use the application consistently [30].

Some participants had low engagement with the “Term-Jai” application. One participant cited technical issues, and two others stated that they could remember the content and perform the exercises without using the application. These findings are consistent with a previous study that demonstrated low adherence to application usage due to technical difficulties and lack of interest [31]. Additionally, after initially engaging with a digital intervention, users might achieve adequate self-regulation, making the application unnecessary [32]. Providing technical support and personalized exercise progression could increase engagement with the application.

The average SUS score was 80%, indicating a good user experience and supporting the “Term-Jai” application’s usability [33]. All participants found the application’s information useful, with most stating it was easy to understand and clear. A qualitative analysis of the interviews provided further insight into the application’s benefits. The mean participant satisfaction score was high, emphasizing its potential as an effective CR tool. Participants gave high ratings to the application’s design, emphasizing the importance of an intuitive and aesthetically pleasing interface. A well-designed application can significantly enhance user experience, making it easier for patients to navigate and access the necessary information [34]. This is particularly relevant for elderly Thai users, who often face challenges in adopting technology due to difficulties navigating complex digital tools [35]. To address this, our application is designed as a simple, user-friendly solution tailored to this demographic, ensuring accessibility and ease of use. However, some technical issues were noted, indicating the need for continuous technical support. Some participants also suggested improvements, such as adding more interactive features and enhancing exercise customization options to better tailor the application to individual recovery needs. Implementing these suggestions could further enhance the application’s utility and user satisfaction.

One of the primary benefits of this feasibility study was improved healthy behaviors. The development of this application aligns with growing evidence supporting the use of mobile health technologies to promote healthy behaviors among individuals at risk of or living with cardiovascular disease [36,37]. The present application contributes to this movement by offering accessible tools to support daily exercise, self-assessment, and comprehensive educational materials on surgery, recovery, potential complications, and lifestyle modifications. Increased patient education is associated with better self-care practices and health outcomes [38,39]. Additionally, gamification was shown to affect health outcomes positively [40]. However, smoking and alcohol consumption did not change after the intervention, as only one participant was a current smoker, and none consumed alcohol.

This study had some limitations. First, the relatively small sample size may restrict the generalizability of the findings; however, the results can serve as preliminary data for future research. The absence of a control group limits the ability to attribute improvements solely to the application. Additionally, open-heart surgery patients were not involved in the application development process; hence, their needs and preferences were not directly incorporated. The male-dominant composition of the sample also presents a limitation, as men have been shown to respond more strongly to perceived usefulness in technology adoption [41]. This demographic distribution may limit generalizability. Additionally, due to time and budget constraints, the application was only developed for the Android platform, was deployed within a single-center setting, and lacked personalization features.

Despite these limitations, the study offers meaningful contributions. While the level of technological innovation may be considered moderate, the strength of this study lies in its integration of functionality with accessibility. It addresses a critical gap by providing structured, guided exercise sessions and essential cardiovascular education through a simple, intuitive interface. These features are particularly significant for older adults or patients with limited health and digital literacy, who are often excluded from high-tech solutions.

Future research should focus on larger patient populations and conduct multi-center randomized controlled trials (RCTs) to validate these findings and explore the long-term impact of the application on clinical outcomes such as blood pressure, cholesterol levels, or physical fitness level. Steps are being taken to develop an iOS-compatible version of the app to broaden accessibility, and to recruit more diverse participants across urban and rural healthcare settings with a balanced representation in terms of gender, age, and comorbidities. In addition, incorporating user feedback will guide enhancements such as exercise customization, interactive features, and improved personalization to better address individual recovery needs. Technical refinements, including offline functionality, performance optimization, and robust user support services (e.g., in-app help centers, AI chatbots, and live support), are essential to ensuring a seamless and user-friendly experience. Future iterations may integrate additional modules, such as dietary tracking, medication adherence support, and mental health resources, offering a more holistic approach to cardiac rehabilitation. Further investigation into the app’s cost-effectiveness and its potential to reduce healthcare utilization is also warranted. Lastly, the integration of AI-driven personalized recommendations presents a promising direction for enhancing patient engagement and outcomes.

## 5. Conclusions

The study demonstrated that the “Term-Jai” smartphone application could be a valuable tool for supporting postoperative care in open-heart surgery patients. High patient satisfaction and positive feedback on the application’s content and design suggest that it could enhance recovery by providing easily accessible information. Addressing barriers and incorporating patient suggestions is crucial for the application’s ongoing development and effectiveness.

## Figures and Tables

**Table 1:** Semi-structured interview guide.

Questions	Additional Instructions
What do you like or dislike about the app?	Why do you like or dislike that part?
What part of the application do you find confusing or difficult to understand?	Can you tell a bit more about that? Why do you think that? How do you have suggestions for improving that parts?
Which part of the application do you think should be improved?	Is the design easy to use, including factors like color, font, and content? Why do you think that? Can you tell me more about that?
What makes you use the application consistently?	Do you use the application more, the same, or less than when you first started, and why? Which part of the app will make you use it consistently? Do you have a willingness to use the app if you do not participate in this study, and why?
What would be the reason for you to stop using the app?	Why do you think that? Do you have any suggestions?
What do you think about the knowledge content in the app?	Is knowledge content easy to understand? Does the knowledge content in the app enhance your understanding of heart disease and post-surgery recovery? Do you have any suggestions?
What do you think about the exercise program in the app?	Is exercise program easy to do? How do instructional exercise videos help you to do the exercise routine? Do you have any suggestions?
What do you think about the exercise tracking, both in terms of a calendar and visual representations illustrating the growth of a tree?	How does exercise tracking help in motivating you to work out? Do you have any suggestions on how to create motivation for exercising?

**Table 2:** Baseline Participants Characteristics.

Characteristic	Value (n = 10)
**Age, years** (**Mean ± SD**)	60.4 ± 10.0
**BMI, (kg/m^2^)** (**Mean ± SD**)	25.0 ± 3.5
**Gender, n (%)**	
Male	8 (80%)
Female	2 (20%)
**Educational Level, n (%)**	
Primary	1 (10%)
Secondary	5 (50%)
Higher	4 (40%)
**Type of open-heart surgery, n (%)**	
CABG	5 (50%)
Valve Replacement	4 (40%)
Others (aortic hemiarch replacement)	1 (10%)
**Comorbidities, n (%)**	
Hypertension	5 (50%)
Dyslipidemia	3 (30%)
Diabetes Mellitus	2 (20%)
Chronic Kidney Disease	0
Chronic Lung Disease	1 (10%)
Others	4 (40%)
**Smoking status, n (%)**	
Nonsmokers	5 (10%)
Former smokers	4 (10%)
Current smokers	1 (10%)

**Table 3:** Usability on the “Term-Jai” application (n = 10).

Category	Score (n = 10)
1. I think that I would like to use this application frequently	4.2 ± 0.8
2. I found the application unnecessarily complex	2.2 ± 0.8
3. I thought the application was easy to use	4.7 ± 0.4
4. I think that I would need the support of a technical person to be able to use this application	2.6 ± 1.3
5. I found the various functions in this application were well integrated	4.2 ± 0.4
6. I thought there was too much inconsistency in this application	1.7 ± 0.8
7. I would imagine that most people would learn to use this application very quickly	4.5 ± 0.5
8. I found the application very cumbersome to use	1.7 ± 0.4
9. I felt very confident using the application	4.5 ± 0.5
10. I needed to learn a lot of things before I could get going with this application	1.8 ± 0.4

Values are presented as mean ± SD. Five-point Likert scale used.

**Table 4:** User satisfaction on “Term-Jai” application (n = 10).

Category	Extremely (n)	Very (n)	Neutral (n)	Slightly (n)	Not at all (n)	Value (Mean ± SD)
**Contents**						
The information is useful to me	5	5	0	0	0	4.5 ± 0.5
The information is clear	4	5	1	0	0	4.3 ± 0.6
Content is easy to understand	6	3	1	0	0	4.5 ± 0.7
Content quantity is appropriate	3	6	1	0	0	4.2 ± 0.6
**Design of the application**						
The application makes it easy to learn	4	4	2	0	0	4.2 ± 0.7
Font size of letter is appropriate	3	5	2	0	0	4.1 ± 0.7
Overall composition of the application is appropriate	2	4	4	0	0	3.8 ± 0.7
Color of the screen is appropriate	4	6	0	0	0	4.4 ± 0.5
**Average**						**4.3 ± 0.6**

**Table 5:** Healthy behaviors (n = 10).

Habits	30-Day Before Intervention	30-Day After Intervention
1. Exercise or engage in physical activities	2.3 ± 1.2	4.8 ± 0.6
2. Increased consumption of vegetables and fruits	1.8 ± 1.0	3.9 ± 0.8
3. Reduced consumption of salt	1.8 ± 1.0	4.5 ± 0.8
4. Reduced high-fat foods	2.0 ± 1.0	4.4 ± 0.5
5. Reduced high-sugar foods	2.1 ± 1.1	4.3 ± 0.8
6. Stress management	3.0 ± 1.2	4.9 ± 0.3
7. Smoking cessation	4.6 ± 1.2	4.7 ± 0.6
8. Reduced alcohol consumption	5.0 ± 0.0	5.0 ± 0.0

**Table 6:** Themes and participant quotes from the semi-structured interview.

Themes	Subthemes	Sample Quotes from Participants
Content of the app is appropriate	Exercise programs are useful	*“Well, it’s not that difficult. The good thing is I can follow the steps. It is very easy and beneficial.”* (74-year-old Male) *“Before the surgery, I didn’t exercise at all and I had no idea how to exercise after surgery. Then I used the app, it’s really good. I’ve learned how to do the exercises.”* (44-year-old Female) *“The exercise program is easy, and it worked for me.”* (60-year-old Male) *“I think the exercise sessions and breathing exercises are good.”* (68-year-old Male)
	Educational sessions are useful	*“It can help me. The knowledge helps me get better.”* (50-year-old Female) *“Well, that’s good, it gives me knowledge that I didn’t know before.”* (57-year-old Male)
		*“It gives good knowledge, good content, clearly explained and easy to understand.”* (51-year-old Male)
	Motivation of exercise from the illustration metaphor	*“Tracking exercise is very applicable. I can see if I did exercise today. The growth of the tree encourages me to exercise.”* (72-year-old Male)
		*“It’s like I added fertilizer to the tree. I was so happy when I saw the tree had grown. It’s fun to keep growing this tree. This made me want to do the exercise every day.”* (78-year-old Male) *“This is good. I saw the growth of the tree and it motivated me to do more exercise.”* (60-year-old Male)
Design of the app is appropriate	Convenience and Ease of Use	*“I’m not confused at all. It’s easy to use and understand.”* (65-year-old Male) *“It’s easy to learn and understand.”* (50-year-old Female) *“Explaining with images and videos is easy to understand and follow.”* (68-year-old Male)
Barriers to use	User-friendly Technical issues	*“I like the app telling me what to do in each step. I just follow that step.”* (74-year-old Female) *“As far as I can tell, it’s nothing complicated in the app. It explains well how to do exercise. Well, it’s easy to understand.”* (44-year-old Female) *“The guided videos help me to do the exercise better.”* (60-year-old Male) *“Sometimes it’s quite slow to response and stutters.”* (60-year-old Male) *“I’m not sure if it’s me or if it’s something that doesn’t work with my device. Sometimes, I can’t find the app.”* (55-year-old Male) *“Um, not sure if it’s the app itself or my own internet, because it lags often, and it freezes frequently. Then I have to pause, then play again. It’s quite annoying.”* (44-year-old Female)
	Familiarity of contents in the app	*“Even without using the app, I can do the exercises because I already remember all the contents”.* (51-year-old Male) *“If the patient has recovered well, it may not be necessary to use the app.”* (60-year-old Male)
Suggestions for the app improvement	Addition of new exercise	*“It could include various forms of exercises; patients might recover better.”* (60-year-old Male) *“Maybe add new types of exercises that patients don’t know about.”* (51-year-old Male)
	Format improvement	*“The app is easy to understand by using pictures. But I think the color is not very pleasing.”* (68-year-old Male)

## Data Availability

The data presented in this study are available on request from the corresponding author. The data are not publicly available due to ethical restrictions related to participant confidentiality and the terms of informed consent.
